# 6-Chloro-8-methyl-4*H*-3,1-benzoxazine-2,4(1*H*)-dione

**DOI:** 10.1107/S1600536810013346

**Published:** 2010-04-21

**Authors:** Yan-Ling Zhou, Hua Wang, Min Zhao

**Affiliations:** a Department of Life Science, Northeast Forestry University, Harbin 150040, People’s Republic of China; b Graduate School of Chinese Academy of Agricultural Sciences, Beijing 100081, People’s Republic of China

## Abstract

The two mol­ecules in the asymmetric unit of the title compound, C_9_H_6_ClNO_3_, are nearly planar, with r.m.s. deviations of 0.034 and 0.037 Å. The crystal structure is stabilized by two weak inter­molecular N—H⋯O inter­actions.

## Related literature

For background to isatoic anhydrides, see: Miyamae (1996 ▶); Nawrot *et al.* (1997 ▶); Nawrot & Sprinz (1998 ▶); Deifel *et al.* (2010 ▶); Ren *et al.* (1996 ▶). For the preparation, see: Coppola (1980 ▶).
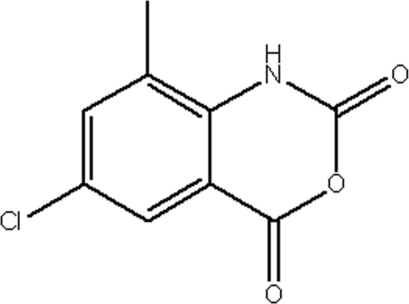


## Experimental

### 

#### Crystal data

C_9_H_6_ClNO_3_
*M* *_r_* = 211.60
Monoclinic, 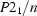

*a* = 8.3019 (12) Å
*b* = 13.1322 (18) Å
*c* = 15.742 (2) Å
β = 99.675 (9)°
*V* = 1691.8 (4) Å^3^
*Z* = 8
Cu *K*α radiation
μ = 3.85 mm^−1^
*T* = 173 K
0.22 × 0.22 × 0.15 mm

#### Data collection

Rigaku R-AXIS RAPID IP area-detector diffractometer
Absorption correction: numerical (*NUMABS*; Higashi, 1999 ▶) *T*_min_ = 0.485, *T*_max_ = 0.596
11524 measured reflections
3050 independent reflections
2280 reflections with *I* > 2σ(*I*)
*R*_int_ = 0.054

#### Refinement

*R*[*F* ^2^ > 2σ(*F* ^2^)] = 0.042
*wR*(*F* ^2^) = 0.107
*S* = 1.01
3050 reflections
256 parameters
H-atom parameters constrained
Δρ_max_ = 0.49 e Å^−3^
Δρ_min_ = −0.29 e Å^−3^

### 

Data collection: *RAPID-AUTO* (Rigaku, 2001 ▶); cell refinement: *RAPID-AUTO*; data reduction: *RAPID-AUTO*; program(s) used to solve structure: *SHELXS97* (Sheldrick, 2008 ▶); program(s) used to refine structure: *SHELXL97* (Sheldrick, 2008 ▶); molecular graphics: *XP* in *SHELXTL* (Sheldrick, 2008 ▶); software used to prepare material for publication: *SHELXL97*.

## Supplementary Material

Crystal structure: contains datablocks I, global. DOI: 10.1107/S1600536810013346/vm2022sup1.cif

Structure factors: contains datablocks I. DOI: 10.1107/S1600536810013346/vm2022Isup2.hkl

Additional supplementary materials:  crystallographic information; 3D view; checkCIF report

## Figures and Tables

**Table 1:** Hydrogen-bond geometry (Å, °)

*D*—H⋯*A*	*D*—H	H⋯*A*	*D*⋯*A*	*D*—H⋯*A*
N1—H1*A*⋯O6^i^	0.88	1.99	2.846 (3)	163
N2—H2*A*⋯O3^ii^	0.88	2.01	2.850 (2)	160

Symmetry codes: (i) 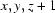; (ii) 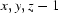.

**Fig. 1.**
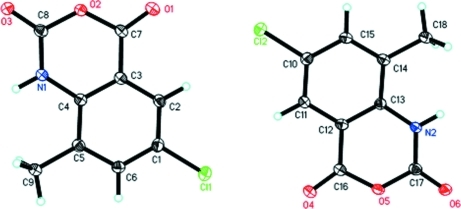
The molecular structure of the title compound showing 50% probability displacement ellipsoids and atom-numbering scheme.

## supplementary crystallographic information

### Comment

Isatoic anhydride derivatives are generally used as intermediates in the synthesis of heterocyclic compounds, such as agricultural chemicals, medicines, pharmaceuticals, quinazolinones, quinazolones, benzimidazolones, phthalimides, pyrroloquinazolones, quinazolinediones and in the fluorescent labeling of mRNAand tRNA (Miyamae, 1996; Nawrot *et al.*, 1997; Nawrot *et al.*, 1998; Deifel *et al.*, 2010; Ren *et al.*, 1996).

The title compound is a nearly planar molecule (Fig. 1). The bond distances are consistent with an aromatic system. There are two molecules in the asymmetric unit of the title compound. The molecular structure is stabilized by two weak intermolecular N–H···O interactions resulting in the formation of dimers.

### Experimental

The isatoic anhydride was prepared by reaction of anthranilic acid with triphosgene in good yield (Coppola, 1980). The title compound (0.2 g) was dissolved in ethanol (50 ml) at room temperature. Colourless blocks of (I) were obtained through slow evaporation after two weeks.

### Refinement

The H atoms were placed at calculated positions, with C—H = 0.93–0.98 Å, and refined as riding with *U*iso(H) = 1.2–1.5*U*eq(C).

### Crystal data

**Table:**

C_9_H_6_ClNO_3_	*F*(000) = 864
*M**_r_* = 211.60	*D*_x_ = 1.661 Mg m^−^^3^
Monoclinic, *P*2_1_/*n*	Cu *K*α radiation, λ = 1.54186 Å
Hall symbol: -P 2yn	Cell parameters from 687 reflections
*a* = 8.3019 (12) Å	θ = 3.1–68.2°
*b* = 13.1322 (18) Å	µ = 3.85 mm^−^^1^
*c* = 15.742 (2) Å	*T* = 173 K
β = 99.675 (9)°	Plate, colorless
*V* = 1691.8 (4) Å^3^	0.22 × 0.22 × 0.15 mm
*Z* = 8	

### Data collection

**Table:**

Rigaku R-AXIS RAPID IP area-detector diffractometer	3050 independent reflections
Radiation source: rotating anode	2280 reflections with *I* > 2σ(*I*)
graphite	*R*_int_ = 0.054
ω scans	θ_max_ = 68.2°, θ_min_ = 4.4°
Absorption correction: numerical (*NUMABS*; Higashi, 1999)	*h* = −10→9
*T*_min_ = 0.485, *T*_max_ = 0.596	*k* = −15→15
11524 measured reflections	*l* = −18→17

### Refinement

**Table:**

Refinement on *F*^2^	Secondary atom site location: difference Fourier map
Least-squares matrix: full	Hydrogen site location: inferred from neighbouring sites
*R*[*F*^2^ > 2σ(*F*^2^)] = 0.042	H-atom parameters constrained
*wR*(*F*^2^) = 0.107	*w* = 1/[σ^2^(*F*_o_^2^) + (0.0569*P*)^2^] where *P* = (*F*_o_^2^ + 2*F*_c_^2^)/3
*S* = 1.01	(Δ/σ)_max_ < 0.001
3050 reflections	Δρ_max_ = 0.49 e Å^−^^3^
256 parameters	Δρ_min_ = −0.29 e Å^−^^3^
0 restraints	Extinction correction: *SHELXL97* (Sheldrick, 2008), Fc^*^=kFc[1+0.001xFc^2^λ^3^/sin(2θ)]^-1/4^
Primary atom site location: structure-invariant direct methods	Extinction coefficient: 0.0023 (3)

### Special details

**Table:**

Geometry. All esds (except the esd in the dihedral angle between two l.s. planes) are estimated using the full covariance matrix. The cell esds are taken into account individually in the estimation of esds in distances, angles and torsion angles; correlations between esds in cell parameters are only used when they are defined by crystal symmetry. An approximate (isotropic) treatment of cell esds is used for estimating esds involving l.s. planes.
Refinement. Refinement of *F*^2^ against ALL reflections. The weighted *R*-factor wR and goodness of fit *S* are based on *F*^2^, conventional *R*-factors *R* are based on *F*, with *F* set to zero for negative *F*^2^. The threshold expression of *F*^2^ > σ(*F*^2^) is used only for calculating *R*-factors(gt) etc. and is not relevant to the choice of reflections for refinement. *R*-factors based on *F*^2^ are statistically about twice as large as those based on *F*, and *R*- factors based on ALL data will be even larger.

### Fractional atomic coordinates and isotropic or equivalent isotropic displacement parameters (Å^2^)

**Table:**

	*x*	*y*	*z*	*U*_iso_*/*U*_eq_	
Cl1	0.74255 (7)	0.37308 (4)	0.31233 (4)	0.03722 (19)	
Cl2	0.15868 (6)	0.36329 (4)	0.20423 (3)	0.03096 (18)	
O1	0.15834 (17)	0.35341 (10)	0.42141 (10)	0.0352 (4)	
O2	0.21462 (17)	0.36049 (9)	0.56311 (10)	0.0292 (4)	
O3	0.25940 (17)	0.36009 (10)	0.70532 (10)	0.0343 (4)	
O4	0.74385 (17)	0.36875 (10)	0.09544 (10)	0.0325 (4)	
O5	0.68671 (17)	0.37641 (10)	−0.04621 (9)	0.0281 (4)	
O6	0.64195 (17)	0.37865 (10)	−0.18829 (10)	0.0342 (4)	
N1	0.4795 (2)	0.37337 (11)	0.63750 (11)	0.0263 (4)	
H1A	0.5472	0.3789	0.6867	0.032*	
N2	0.4212 (2)	0.38050 (11)	−0.12090 (11)	0.0248 (4)	
H2A	0.3531	0.3836	−0.1701	0.030*	
C1	0.6645 (3)	0.37491 (14)	0.40801 (14)	0.0287 (5)	
C2	0.4986 (3)	0.36648 (13)	0.40569 (14)	0.0265 (5)	
H2B	0.4261	0.3607	0.3524	0.032*	
C3	0.4396 (3)	0.36668 (13)	0.48353 (14)	0.0251 (5)	
C4	0.5457 (3)	0.37476 (13)	0.56177 (13)	0.0232 (5)	
C5	0.7156 (3)	0.38488 (13)	0.56459 (14)	0.0255 (5)	
C6	0.7711 (3)	0.38478 (13)	0.48592 (14)	0.0270 (5)	
H6A	0.8849	0.3916	0.4854	0.032*	
C7	0.2637 (3)	0.35950 (14)	0.48294 (14)	0.0262 (5)	
C8	0.3186 (3)	0.36409 (14)	0.64046 (15)	0.0277 (5)	
C9	0.8305 (2)	0.39789 (15)	0.64787 (13)	0.0297 (5)	
H9A	0.9427	0.4032	0.6365	0.045*	
H9B	0.8217	0.3390	0.6851	0.045*	
H9C	0.8023	0.4600	0.6766	0.045*	
C10	0.2366 (3)	0.37177 (13)	0.10859 (14)	0.0260 (5)	
C11	0.4028 (2)	0.36882 (13)	0.11139 (14)	0.0250 (5)	
H11A	0.4754	0.3641	0.1648	0.030*	
C12	0.4623 (3)	0.37296 (13)	0.03310 (14)	0.0245 (5)	
C13	0.3552 (3)	0.37864 (12)	−0.04491 (13)	0.0226 (5)	
C14	0.1853 (2)	0.38394 (14)	−0.04755 (14)	0.0248 (5)	
C15	0.1296 (3)	0.38043 (12)	0.03084 (14)	0.0249 (5)	
H15A	0.0155	0.3841	0.0313	0.030*	
C16	0.6380 (3)	0.37193 (13)	0.03436 (14)	0.0252 (5)	
C17	0.5828 (3)	0.37785 (14)	−0.12356 (15)	0.0278 (5)	
C18	0.0708 (2)	0.39453 (15)	−0.13148 (14)	0.0299 (5)	
H18A	−0.0421	0.3958	−0.1208	0.045*	
H18B	0.0852	0.3367	−0.1688	0.045*	
H18C	0.0944	0.4580	−0.1597	0.045*	

### Atomic displacement parameters (Å^2^)

**Table:**

	*U*^11^	*U*^22^	*U*^33^	*U*^12^	*U*^13^	*U*^23^
Cl1	0.0344 (3)	0.0529 (4)	0.0255 (3)	0.0024 (2)	0.0083 (3)	−0.0006 (2)
Cl2	0.0292 (3)	0.0399 (3)	0.0246 (3)	−0.0011 (2)	0.0072 (2)	0.0001 (2)
O1	0.0251 (8)	0.0467 (9)	0.0321 (10)	−0.0004 (7)	−0.0004 (8)	0.0003 (7)
O2	0.0202 (7)	0.0424 (9)	0.0244 (9)	−0.0006 (6)	0.0020 (7)	0.0030 (6)
O3	0.0242 (8)	0.0540 (10)	0.0252 (9)	−0.0013 (7)	0.0053 (7)	0.0004 (7)
O4	0.0222 (8)	0.0449 (9)	0.0287 (9)	0.0000 (6)	−0.0012 (8)	0.0026 (7)
O5	0.0178 (7)	0.0407 (8)	0.0252 (9)	−0.0001 (6)	0.0019 (7)	−0.0038 (6)
O6	0.0220 (8)	0.0575 (10)	0.0240 (9)	−0.0022 (6)	0.0062 (7)	−0.0037 (7)
N1	0.0210 (9)	0.0348 (10)	0.0225 (10)	−0.0011 (7)	0.0016 (8)	−0.0008 (7)
N2	0.0177 (9)	0.0349 (9)	0.0209 (10)	−0.0006 (7)	0.0007 (8)	0.0000 (7)
C1	0.0324 (12)	0.0304 (11)	0.0236 (13)	0.0030 (9)	0.0059 (10)	0.0010 (8)
C2	0.0259 (11)	0.0275 (11)	0.0250 (12)	0.0030 (8)	0.0007 (10)	0.0010 (8)
C3	0.0236 (11)	0.0251 (10)	0.0257 (12)	0.0007 (8)	0.0015 (10)	0.0004 (8)
C4	0.0228 (11)	0.0213 (10)	0.0249 (12)	0.0001 (7)	0.0025 (10)	0.0008 (8)
C5	0.0244 (11)	0.0245 (10)	0.0268 (13)	0.0015 (8)	0.0021 (10)	−0.0015 (8)
C6	0.0222 (11)	0.0300 (11)	0.0289 (13)	0.0005 (8)	0.0047 (10)	−0.0005 (9)
C7	0.0232 (11)	0.0278 (11)	0.0258 (13)	−0.0001 (8)	−0.0015 (10)	0.0006 (8)
C8	0.0247 (11)	0.0280 (11)	0.0292 (13)	0.0015 (8)	0.0009 (10)	0.0037 (9)
C9	0.0222 (11)	0.0388 (12)	0.0272 (13)	−0.0023 (9)	0.0019 (10)	−0.0019 (9)
C10	0.0275 (11)	0.0256 (10)	0.0253 (13)	−0.0024 (8)	0.0060 (10)	−0.0020 (8)
C11	0.0230 (11)	0.0282 (11)	0.0230 (12)	0.0003 (8)	0.0012 (10)	0.0004 (8)
C12	0.0206 (10)	0.0240 (10)	0.0284 (13)	−0.0015 (8)	0.0028 (10)	−0.0013 (8)
C13	0.0231 (11)	0.0223 (10)	0.0224 (12)	−0.0011 (8)	0.0037 (10)	0.0001 (8)
C14	0.0206 (10)	0.0240 (10)	0.0288 (13)	−0.0004 (8)	0.0011 (10)	−0.0003 (8)
C15	0.0197 (10)	0.0259 (10)	0.0286 (13)	−0.0006 (8)	0.0030 (9)	−0.0007 (8)
C16	0.0219 (11)	0.0261 (10)	0.0265 (13)	0.0006 (8)	0.0012 (10)	−0.0006 (8)
C17	0.0250 (11)	0.0300 (11)	0.0277 (13)	−0.0018 (8)	0.0022 (10)	−0.0039 (9)
C18	0.0205 (11)	0.0381 (12)	0.0304 (13)	0.0020 (9)	0.0020 (10)	0.0008 (9)

### Geometric parameters (Å, °)

**Table:**

Cl1—C1	1.737 (2)	C3—C7	1.462 (3)
Cl2—C10	1.739 (2)	C4—C5	1.410 (3)
O1—C7	1.194 (2)	C5—C6	1.392 (3)
O2—C8	1.370 (3)	C5—C9	1.496 (3)
O2—C7	1.390 (3)	C6—H6A	0.9500
O3—C8	1.206 (2)	C9—H9A	0.9800
O4—C16	1.189 (3)	C9—H9B	0.9800
O5—C17	1.369 (3)	C9—H9C	0.9800
O5—C16	1.396 (3)	C10—C11	1.374 (3)
O6—C17	1.203 (2)	C10—C15	1.391 (3)
N1—C8	1.350 (3)	C11—C12	1.403 (3)
N1—C4	1.394 (3)	C11—H11A	0.9500
N1—H1A	0.8800	C12—C13	1.392 (3)
N2—C17	1.349 (3)	C12—C16	1.456 (3)
N2—C13	1.397 (3)	C13—C14	1.406 (3)
N2—H2A	0.8800	C14—C15	1.389 (3)
C1—C2	1.376 (3)	C14—C18	1.499 (3)
C1—C6	1.393 (3)	C15—H15A	0.9500
C2—C3	1.394 (3)	C18—H18A	0.9800
C2—H2B	0.9500	C18—H18B	0.9800
C3—C4	1.393 (3)	C18—H18C	0.9800
			
C8—O2—C7	124.77 (17)	H9A—C9—H9B	109.5
C17—O5—C16	124.97 (17)	C5—C9—H9C	109.5
C8—N1—C4	124.42 (19)	H9A—C9—H9C	109.5
C8—N1—H1A	117.8	H9B—C9—H9C	109.5
C4—N1—H1A	117.8	C11—C10—C15	121.3 (2)
C17—N2—C13	124.13 (18)	C11—C10—Cl2	119.22 (17)
C17—N2—H2A	117.9	C15—C10—Cl2	119.45 (17)
C13—N2—H2A	117.9	C10—C11—C12	118.0 (2)
C2—C1—C6	121.1 (2)	C10—C11—H11A	121.0
C2—C1—Cl1	119.58 (18)	C12—C11—H11A	121.0
C6—C1—Cl1	119.34 (17)	C13—C12—C11	120.68 (19)
C1—C2—C3	118.3 (2)	C13—C12—C16	120.2 (2)
C1—C2—H2B	120.8	C11—C12—C16	119.14 (19)
C3—C2—H2B	120.8	C12—C13—N2	118.17 (19)
C4—C3—C2	120.9 (2)	C12—C13—C14	121.2 (2)
C4—C3—C7	119.6 (2)	N2—C13—C14	120.65 (19)
C2—C3—C7	119.51 (19)	C15—C14—C13	116.99 (19)
C3—C4—N1	118.26 (19)	C15—C14—C18	121.99 (19)
C3—C4—C5	121.1 (2)	C13—C14—C18	121.01 (19)
N1—C4—C5	120.68 (19)	C14—C15—C10	121.7 (2)
C6—C5—C4	116.81 (19)	C14—C15—H15A	119.1
C6—C5—C9	121.48 (19)	C10—C15—H15A	119.1
C4—C5—C9	121.69 (19)	O4—C16—O5	116.66 (19)
C5—C6—C1	121.8 (2)	O4—C16—C12	127.9 (2)
C5—C6—H6A	119.1	O5—C16—C12	115.46 (18)
C1—C6—H6A	119.1	O6—C17—N2	125.1 (2)
O1—C7—O2	116.79 (19)	O6—C17—O5	117.84 (19)
O1—C7—C3	127.2 (2)	N2—C17—O5	117.00 (19)
O2—C7—C3	116.02 (18)	C14—C18—H18A	109.5
O3—C8—N1	125.4 (2)	C14—C18—H18B	109.5
O3—C8—O2	117.74 (19)	H18A—C18—H18B	109.5
N1—C8—O2	116.9 (2)	C14—C18—H18C	109.5
C5—C9—H9A	109.5	H18A—C18—H18C	109.5
C5—C9—H9B	109.5	H18B—C18—H18C	109.5
			
C6—C1—C2—C3	−0.8 (3)	C15—C10—C11—C12	−1.1 (3)
Cl1—C1—C2—C3	179.08 (13)	Cl2—C10—C11—C12	178.26 (12)
C1—C2—C3—C4	−0.3 (3)	C10—C11—C12—C13	−0.7 (3)
C1—C2—C3—C7	179.00 (16)	C10—C11—C12—C16	178.83 (16)
C2—C3—C4—N1	−179.29 (15)	C11—C12—C13—N2	−178.74 (15)
C7—C3—C4—N1	1.4 (2)	C16—C12—C13—N2	1.7 (2)
C2—C3—C4—C5	1.2 (3)	C11—C12—C13—C14	2.2 (3)
C7—C3—C4—C5	−178.09 (17)	C16—C12—C13—C14	−177.37 (16)
C8—N1—C4—C3	0.4 (3)	C17—N2—C13—C12	−0.5 (3)
C8—N1—C4—C5	179.93 (17)	C17—N2—C13—C14	178.58 (16)
C3—C4—C5—C6	−0.9 (3)	C12—C13—C14—C15	−1.7 (3)
N1—C4—C5—C6	179.55 (15)	N2—C13—C14—C15	179.27 (15)
C3—C4—C5—C9	177.47 (16)	C12—C13—C14—C18	177.37 (16)
N1—C4—C5—C9	−2.0 (3)	N2—C13—C14—C18	−1.7 (3)
C4—C5—C6—C1	−0.2 (3)	C13—C14—C15—C10	−0.2 (3)
C9—C5—C6—C1	−178.58 (16)	C18—C14—C15—C10	−179.25 (16)
C2—C1—C6—C5	1.1 (3)	C11—C10—C15—C14	1.6 (3)
Cl1—C1—C6—C5	−178.84 (14)	Cl2—C10—C15—C14	−177.76 (13)
C8—O2—C7—O1	178.00 (15)	C17—O5—C16—O4	178.23 (15)
C8—O2—C7—C3	−2.3 (2)	C17—O5—C16—C12	−2.5 (2)
C4—C3—C7—O1	179.09 (18)	C13—C12—C16—O4	178.89 (18)
C2—C3—C7—O1	−0.2 (3)	C11—C12—C16—O4	−0.7 (3)
C4—C3—C7—O2	−0.5 (2)	C13—C12—C16—O5	−0.3 (2)
C2—C3—C7—O2	−179.82 (15)	C11—C12—C16—O5	−179.91 (14)
C4—N1—C8—O3	177.97 (17)	C13—N2—C17—O6	179.44 (17)
C4—N1—C8—O2	−3.1 (3)	C13—N2—C17—O5	−2.1 (3)
C7—O2—C8—O3	−176.85 (16)	C16—O5—C17—O6	−177.76 (16)
C7—O2—C8—N1	4.1 (2)	C16—O5—C17—N2	3.6 (3)

### Hydrogen-bond geometry (Å, °)

**Table:**

*D*—H···*A*	*D*—H	H···*A*	*D*···*A*	*D*—H···*A*
N1—H1A···O6^i^	0.88	1.99	2.846 (3)	163
N2—H2A···O3^ii^	0.88	2.01	2.850 (2)	160

Symmetry codes: (i) *x*, *y*, *z*+1; (ii) *x*, *y*, *z*−1.
